# Population genomics reveals demographic history and selection signatures of hazelnut (*Corylus*)

**DOI:** 10.1093/hr/uhad065

**Published:** 2023-04-10

**Authors:** Zhen Yang, Wenxu Ma, Lujun Wang, Xiaohong Yang, Tiantian Zhao, Lisong Liang, Guixi Wang, Qinghua Ma

**Affiliations:** Key Laboratory of Tree Breeding and Cultivation, National Forestry and Grassland Administration, Research Institute of Forestry, Chinese Academy of Forestry, Beijing, 100091, China; Key Laboratory of Tree Breeding and Cultivation, National Forestry and Grassland Administration, Research Institute of Forestry, Chinese Academy of Forestry, Beijing, 100091, China; Forest Botany and Tree Physiology, University of Goettingen, Goettingen, 37077, Germany; Research Institute of Economic Forest Cultivation and Processing, Anhui Academy of Forestry, Hefei, 230031, China; Research Institute of Walnut, Guizhou Academy of Forestry, Guiyang, 550005, China; Key Laboratory of Tree Breeding and Cultivation, National Forestry and Grassland Administration, Research Institute of Forestry, Chinese Academy of Forestry, Beijing, 100091, China; Key Laboratory of Tree Breeding and Cultivation, National Forestry and Grassland Administration, Research Institute of Forestry, Chinese Academy of Forestry, Beijing, 100091, China; Key Laboratory of Tree Breeding and Cultivation, National Forestry and Grassland Administration, Research Institute of Forestry, Chinese Academy of Forestry, Beijing, 100091, China; Key Laboratory of Tree Breeding and Cultivation, National Forestry and Grassland Administration, Research Institute of Forestry, Chinese Academy of Forestry, Beijing, 100091, China

## Abstract

Hazelnut (*Corylus* spp.) is known as one of the four famous tree nuts in the world due to its pleasant taste and nutritional benefits. However, hazelnut promotion worldwide is increasingly challenged by global climate change, limiting its production to a few regions. Focusing on the eurytopic Section *Phyllochlamys*, we conducted whole-genome resequencing of 125 diverse accessions from five geo-ecological zones in Eurasia to elucidate the genomic basis of adaptation and improvement. Population structure inference outlined five distinct genetic lineages corresponding to climate conditions and breeding background, and highlighted the differentiation between European and Asian lineages. Demographic dynamics and ecological niche modeling revealed that Pleistocene climatic oscillations dominantly shaped the extant genetic patterns, and multiple environmental factors have contributed to the lineage divergence. Whole-genome scans identified 279, 111, and 164 selective sweeps that underlie local adaptation in *Corylus heterophylla*, *Corylus kweichowensis*, and *Corylus yunnanensis*, respectively. Relevant positively selected genes were mainly involved in regulating signaling pathways, growth and development, and stress resistance. The improvement signatures of hybrid hazelnut were concentrated in 312 and 316 selected genes, when compared to *C. heterophylla* and *Corylus avellana*, respectively, including those that regulate protein polymerization, photosynthesis, and response to water deprivation. Among these loci, 22 candidate genes were highly associated with the regulation of biological quality. Our study provides insights into evolutionary processes and the molecular basis of how sibling species adapt to contrasting environments, and offers valuable resources for future climate-resilient breeding.

## Introduction

Hazelnut, the *Corylus* L. genus within Betulaceae, includes approximately 17 polymorphic deciduous tree and shrub species that produce healthful nuts and oils [1–3]. The genus demonstrates wide morphological diversity and environmental adaptability, with species adapted to forest habitats throughout the north temperate zone [4–6]. Hazelnut is known as one of the four famous tree nuts in the world and has important economic, nutritional, and ecological values. Multilocus phylogenetics revealed that *Corylus* originated in southwest China during the middle Eocene and has spread to Europe and North America through two long-distance dispersal routes: the Himalayan-Central Asia Corridor and Beringian Land Bridge [7]. In the process of evolution, ancestral taxa from different geographical regions have undergone long-term natural selection to adapt to various habitats. Moreover, some taxa have also been subject to recent artificial selection to meet diverse breeding targets.

To date, four sections (*Acanthochlamys*, *Siphonochlamys*, *Phyllochlamys*, and *Colurnae*) have been identified within *Corylus* [5, 8, 9], of which Section *Phyllochlamys* has long been the source of hazelnut breeding because of its superior agronomic traits such as open nuts without bracts completely enclosed and easy artificial propagation. Traditionally, the section is widely recognized to contain three intercontinental disjunct taxa: European hazelnut (*Corylus avellana*), American hazelnut (*Corylus americana*), and Asian hazelnut (*Corylus heterophylla* species complex) [5]. European hazelnut, the solely domesticated *Corylus* species, is distributed in the Europe and Mediterranean region, while American hazelnut occurs only in eastern North America. Asian hazelnut complex is widely distributed in East Asia and shows high genetic diversity, in which three cryptic species (*C. heterophylla*, *Corylus kweichowensis*, and *Corylus yunnanensis*) were further proposed [10, 11]. These series of species share some similar features (e.g. shrubs with leafy involucres and bell-shaped bracts), but present apparent discrepancies in aspects of their morphology, geographic distribution, and ecological niche [12, 13]. Due to recent divergence and introgressive hybridization, genetic boundary and evolutionary relationships within the section have not been clearly clarified by traditional molecular markers such as nuclear internal transcribed spacer (ITS) sequences [5, 14], complete chloroplast genomes [7], and nuclear single-copy genes [1]. These species are either difficult to separate in the nuclear phylogenetic tree or cluster with other distant species due to chloroplast capture. Moreover, the emergence of a large number of hybrid cultivars in the process of crossbreeding and improvement, including the cases in East Asia (*C. avellana* × *C. heterophylla*) [15] and North America (*C. avellana* × *C. americana*) [16], has made the classification of Section *Phyllochlamys* more complex.

Current hazelnut breeding programs mainly emphasize climate adaptation, and a fundamental biological question that has recently emerged concerns how hazelnut adapt to regional environments. In the long evolutionary process, these closely related taxa (i.e. *C. avellana*, *C. americana*, and *C. heterophylla* species complex) of Section *Phyllochlamys* have formed unique adaptation to different continental environments, making them the main body of hazelnut breeding in the world. This local adaptation triggered by divergent selection [17, 18] can lead to responses to various geographically specific selection pressures such as climate, pathogens, photoperiod, and soil property [19, 20]. After long generations of selection and domestication, *C. avellana* have become the most commercially valuable *Corylus* species around the world; however, it is confined to Mediterranean-like climates (e.g. Mediterranean coast of Europe and California in North America) that allow consistent yields. Unsurprisingly, the promotion of *C. avellana* is restricted in non-native cultivation regions, including in mainland China and eastern North America, mainly due to climate inadaptation and severe pathogens [21]. In mainland China, the natural habitats of *C. heterophylla* species complex spans a broad range of growing zones, climates, and soil types, suggesting that the complex holds diverse sources of stress resistance and climate adaptation [22]. The three geo-ecotypes of *C. heterophylla* complex occur successively along the altitudinal and hydrothermal gradients, forming their suitable regions in northern, central and eastern, and southwest China, respectively. However, their morphology deviates somewhat from commercial standards, such as husk enclosed shells, thick shells, and small kernels. Fortunately, interspecific hybridization between *C. heterophylla* and *C. avellana* occurs very readily, generating excellent hybrid cultivars that not only inherit the commercial traits of *C. avellana*, but also expand more suitable regions than *C. heterophylla*. Therefore, Section *Phyllochlamys* is an excellent system for illuminating the genetic basis of evolutionary adaptation at the intercontinental scale. However, traditional molecular markers (i.e. microsatellites, nuclear DNA, and chloroplast DNA) only produce a few variable loci, and so are generally insufficient to characterize the genetic structure of species with complex demographic history and adaptive pattern [23–25].

Recently, population genomics approaches have provided powerful toolsets for the studies of speciation and adaptation. First, population studies at the genomic level greatly increase the resolution and power of traditional genetic approaches, which can be applied to delineate tricky taxa within species complexes [26–28]. Second, with the advance of coalescent statistical models, we can quantitatively infer the demographic history, including trajectory of effective population size, direction, and extent of introgression, as well as divergence time between species/populations [29–31]. Third, the evolutionary process of a population can be reflected by its genetic composition; thus, the footprints of adaptation, domestication, and improvement should be contained in the genomes. This is particularly important because genome-wide scanning of selection has become possible, enabling us to identify genetic variation associated with adaptation and economic traits [32–34]. However, population genomics of *Corylus* has been hindered due to the lack of a reference genome and appropriate populations in previous researches. Fortunately, major achievements have been made in decoding genomes of Section *Phyllochlamys* and its relatives, including *C. heterophylla* [35, 36], *C. avellana* [37], and *Corylus mandshurica* [38]. Exploring the genomic variations based on these genomic resources will promote a wide range of studies (e.g. taxonomy, evolution, and adaptation) that can contribute to resource exploitation in hazelnut breeding.

In order to explore genomic divergence, demographic history, and selection signatures, we here re-sequenced 125 representative accessions of Section *Phyllochlamys*, including Asia hazelnut complex (*C. heterophylla*, *C. kweichowensis*, and *C. yunnanensis*), European hazelnut (*C. avellana*), and hybrids of *C. avellana* × *C. heterophylla*. Specifically, we aim to address the following questions: (i) characterize the genomic diversity and differentiation level among different genetic lineages; (ii) clarify the evolutionary history of *C. heterophylla* complex and explore the genetic footprints involved in geographic adaptation; (iii) capture the selection signals in *C. avellana* and hybrid hazelnut during adaptation and improvement.

## Results

### Population resequencing and genomic variation

We generated the first genomic variation dataset for hazelnut using a collection of 125 diverse accessions within Section *Phyllochlamys*, including 76 Asian hazelnut (32 *C. heterophylla*, 25 *C. kweichowensis*, and 19 *C. yunnanensis*), 12 European hazelnut, and 37 hybrids of Asian and European hazelnuts (Fig. Figure S1; Table S1, see online supplementary material). Based on population resequencing, we obtained 1419 Gb raw data, with average mapping rate of 93.4%, sequencing depth of 26.6×, and coverage of 90.4% on the *C. heterophylla* reference genome. The mapping rate varied slightly in *C. avellana* (94.5%), *C. heterophylla* (95.5%), hybrid hazelnut (95.1%), *C. kweichowensis* (93.6%), and *C. yunnanensis* (85.8%), respectively. The visible differences in mapping rates were mainly caused by the reference genome and divergence among sequenced genotypes (Table S1, see online supplementary material). In total, a final set of 7 177 893 high-quality SNPs were generated in the 125 sequenced hazelnut accessions, with an average of 18.9 SNPs per kilobase. The distribution of SNPs varied greatly among chromosomes (Fig. 2A). More than half of the SNPs (54.20%) were located in intergenic regions, 27.73% were situated in genic regions, and the rest 18.07% were distributed in upstream or downstream regions. Within genic regions, 1 572 487 (21.91%) SNPs were in intronic regions and 417 512 (5.82%) were in exonic regions, of which 209 444 (2.92%) caused amino acid mutations such as non-synonymous substitution, stop-gain and stop-lost. Moreover, 209 444 non-synonymous SNPs and 203 578 synonymous were discovered in the CDS regions, respectively, resulting in a nonsynonymous/synonymous substitution ratio of 1.03 (Table S2, see online supplementary material).

### Phylogenetic inference and population structure

**Figure 1:**
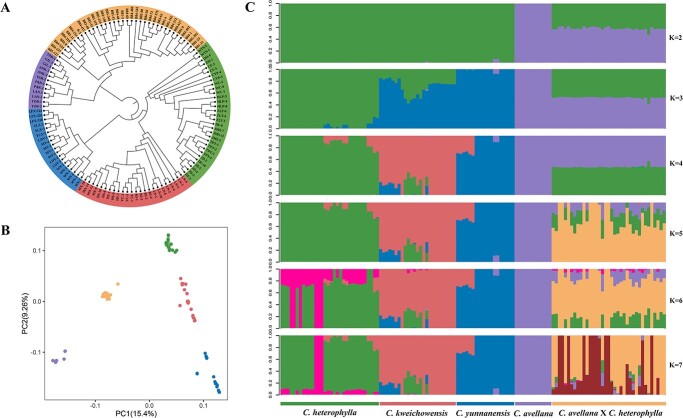
Phylogenetic tree and population structure of hazelnut accessions. **A** Maximum likelihood (ML) tree of 125 accessions constructed based on the LD-pruned SNP dataset. **B** Principal component plots with the first two principal components. Colors correspond to the phylogenetic groups. **C** Population stratification analysis with different number of groups (*K* = 2 to 7). The x axis displays the five groups and the y axis quantifies ancestry memberships. Each color represents one putative ancestral background.

To explore the genetic relationships among 125 hazelnut accessions, phylogenomic inference, principal component analysis, and admixture analysis were performed. The maximum likelihood (ML) tree indicated that these 125 accessions could be divided into five species-specific clades (Fig. 1A), which were basically congruent with their geographic origin and breeding background. The three geo-ecospecies of the *C. heterophylla* complex were clearly separated, of which *C. heterophylla* and *C. kweichowensis* showed a closer sisterhood. Hybrid hazelnut (*C. avellana* × *C. heterophylla*) was more closely to its paternal species (*C. avellana*) than to maternal species (*C. heterophylla*). Principal component analysis (PCA) also illustrated five distinct groups observed in the ML tree, and the first two axes accounted for 15.40% and 9.26% of the total variance, respectively. PC1 clearly distinguished the hybrid combination (*C. avellana*, *C. heterophylla*, and hybrid hazelnut), and PC2 further separated three geo-ecotypes of Asian *C. heterophylla* complex (Fig. 1B).

ADMIXTURE analysis revealed significant population division and extensive admixture among different groups. In the scenario of *K* = 2, *C. heterophylla* complex and *C. avellana* clustered into two distinct groups, while hybrid hazelnut contained a mixture of genetic components of the former two groups (Fig. 1C). When *K* = 3, *C. heterophylla* complex split into two genetic clusters, and when *K* = 4, the rudiment of speciation was basically formed with the expectation of hybrid hazelnut. Five optimal groups were identified according to the lowest CV error when *K* = 5 (Fig. Figure S2, see online supplementary material), which corresponded roughly to *C. avellana*, *C. heterophylla*, *C. kweichowensis*, *C. yunnanensis*, and hybrid hazelnut. Under this scenario, hybrid hazelnut was designated as a distinct group, although it was composed of three genetic components. As *K* increased (6, 7), more complex admixture scenarios occurred within *C. heterophylla* and hybrid hazelnut.

### Genetic diversity, differentiation, and LD decay

**Figure 2:**
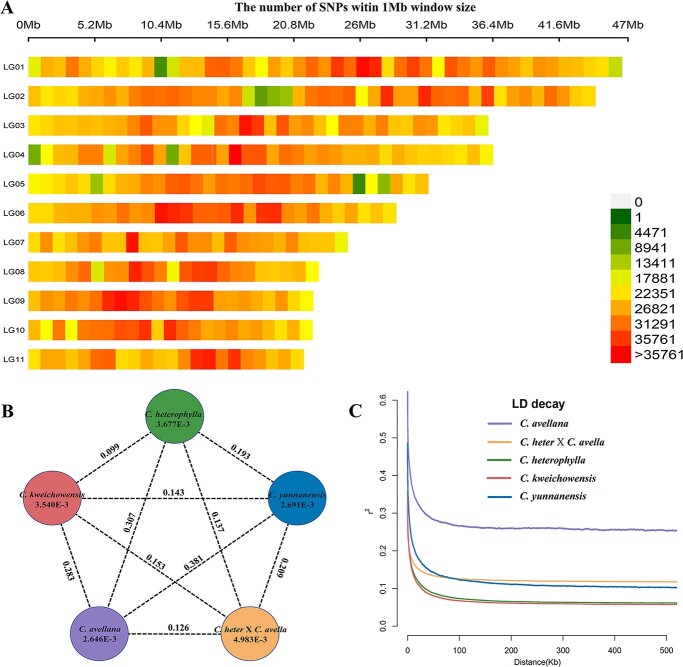
SNP density map, genetic diversity, and linkage disequilibrium patterns. **A** Close-up view of SNP density on each chromosome. **B** Nucleotide diversity and genetic divergence across the five clades of hazelnut accessions. The value in each circle represents a measure of nucleotide diversity (π) for the clade, and the value on each line indicates genetic divergence (*F*_ST_) between two clades. **C** LD decay of the five clades measured by *r*^2^ against distance.

We calculated parameters of heterozygosity, π and *F*_ST_ to evaluate patterns of genomic diversity and genetic divergence among the five genetic lineages. The heterozygosity ranged from 0.334% to 0.855% across all accessions. As expected, hybrid hazelnut harbored the highest heterozygosity (0.822 ± 0.046%), whereas *C. yunnanensis* exhibited the lowest heterozygosity (0.429 ± 0.060%). *C. heterophylla* (0.508 ± 0.075%), *C. kweichowensis* (0.482 ± 0.059%), and *C. avellana* (0.479 ± 0.024%) showed very close and moderate heterozygosity (Table S1, see online supplementary material). In accordance with the results of heterozygosity, hybrid hazelnut had the highest nucleotide diversity (π = 4.983 × 10^−3^), followed by *C. heterophylla* (π = 3.677 × 10^−3^) and *C. kweichowensis* (π = 3.540 × 10^−3^). *C. yunnanensis* (π = 2.691 × 10^−3^) and *C. avellana* (π = 2.646 × 10^−3^) had similar but low nucleotide diversity (Fig. 2B). Overall, the diversity level was roughly consistent with their breeding background, natural distribution, and domestication history: hybridization enhanced the nucleotide diversity of hybrid hazelnut; eurytopic distribution favored *C. heterophylla* and *C. kweichowensis* to maintain similar and moderate diversity, whereas narrow distribution limited the diversity of *C. yunnanensis*; long generations of selection and domestication reduced the diversity of *C. avellana*.

Pairwise *F*-statistics among the five genetic lineages were calculated. At the species level, clear genetic differentiation was observed among the five lineages, with the highest differentiation occurring between *C. avellana* and *C. yunnanensis* (*F*_ST_ = 0.381). Within *C. heterophylla* complex, *C. heterophylla* and *C. kweichowensis* showed the lowest differentiation (*F*_ST_ = 0.099), whereas *C. yunnanensis* displayed relatively high differentiation with the former two (*F*_ST_ = 0.193 and 0.143), which further supported their evolutionary relationships in the phylogenetic tree. Within the hybrid combination, the differentiation level of hybrid hazelnut with its male parent (*C. avellana*) (*F*_ST_ = 0.126) was slightly lower than that with its female parent (*C. heterophylla*) (*F*_ST_ = 0.137) (Fig. 2B). At the genome-wide level, the distribution patterns of *F*_ST_ values of paired species comparisons varied greatly across the genome, with the highly differentiated regions randomly distributed in different regions of 11 chromosomes (Figs 5 and 6; Figs S6–S9, see online supplementary material). The top 5% baseline of the *F*_ST_ empirical distribution was consistent with the species differentiation level, among which *C. avellana* vs *C. heterophylla* showed the highest threshold of 0.685, whereas *C. heterophylla* vs *C. kweichowensis* (0.301), *C. avellana* vs hybrid hazelnut (0.289), *C. heterophylla* vs hybrid hazelnut (0.268) had low and similar thresholds.

Linkage disequilibrium (LD) decay of a population can be affected by many factors, including genetic drift, mutation, selection, recombination, population structure, etc. Thus, population attributes, such as generation length, mating system, natural or domesticated population, and selection intensity, are closely related to the state of LD decay. We observed decreased signatures of genome-wide LD (estimated by *r*^2^) in all the five genetic groups (Fig. 2C). *C. avellana* showed the highest LD level and the slowest decay rate (19.6 kb) to half of the maximum *r*^2^ (0.326), which is in accord with the fact that the species has experienced long generations of selection and domestication in Europe and Mediterranean regions. By contrast, *C. kweichowensis* had the lowest LD level and the fastest decay rate (2.9 kb), which was comparable to that of *C. heterophylla* (3.8 kb) at the same *r*^2^ threshold (~0.19). The similar LD pattern between the two (both are natural populations) is consistent with their low differentiation level and similar nucleotide diversity. The LD level and decay distance (4.9 kb) of *C. yunnanensis* were significantly higher than those of *C. kweichowensis*, although they showed the lowest genetic differentiation. This difference is supposed to be caused by the strong selection pressure exerted by the unique environment in southwest China. As expected, the LD level and decay distance (8.2 kb) of hybrid hazelnut were between the two parents (*C. avellana* and *C. heterophylla*).

### Demographic history and gene flow

**Figure 3:**
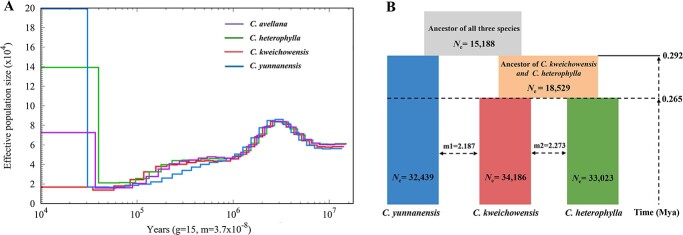
Demographic history and gene flow of the four hazelnut clades. **A** Changes in effective population size (*N*_e_) through time inferred from the PSMC method. **B** Schematic representation of the best-fitting demographic model inferred by ∂a∂i. Widths of the boxes represent the relative *N*_e_ and arrows indicate effective migration per generation between clades to estimate gene flow. The inferred demographic parameters obtained from this model were provided in Table S3 (see online supplementary material).

We performed the pairwise sequentially Markovian coalescent (PSMC) analyses to trace historical trajectory in effective population size (*N*_e_) of four evolutionary lineages (*C. avellana*, *C. heterophylla*, *C. kweichowensis*, and *C. yunnanensis*) (Fig. 3A). The result showed that the ancestors of Section *Phyllochlamys* could date back to approximately 10.0 Mya. After an expansion plateau at about 3.0 Mya, the *N*_e_ value experienced a dramatic and continuous decline until ∼0.8 Mya. Subsequently, *C. yunnanensis*, the high-altitude lineage in southwest China, underwent a more rapid decline than other three lineages (*C. avellana*, *C. heterophylla*, and *C. kweichowensis*) during 0.8–0.1 Mya, with the divergence peak occurring at about 0.3 Mya. By contrast, the latter three lineages exhibited similar historical trajectories until 0.1 Mya, implying a more recent divergence. Although the PSMC profiles ostensibly displayed significant population changes within the last approximately 3000 generations, it could be implausible because PSMC has difficulties in evaluating very recent demographic dynamics.

We further employed ∂a∂i to simulate recent demographic history and gene flow within *C. heterophylla* complex, and 17 three-population models with different divergence scenes, gene flow, and varying population sizes were tested (Fig. S3, see online supplementary material). The residuals illustrated that the model-simulated SFS was quite compatible with the real SFS in all pairwise comparisons (Fig. S4, see online supplementary material), indicating the accuracy of the model and extrapolation results. The maximum log-likelihood value and Akaike information criterion indicated that the best-supported model was ‘adjacent secondary contact with symmetric migration after shorter isolation’ (log-likelihood = −418 444, AIC = 836 904; Table S3, see online supplementary material). This demographic model suggested that *C. yunnanensis* firstly split from the common ancestor at around 0.29 Mya, whereas *C. kweichowensis* and *C. heterophylla* diverged from each other soon afterwards at around 0.26 Mya. Furthermore, our simulations revealed a transient interruption of gene flow between *C. yunnanensis* and ancestors of *C. kweichowensis* and *C. heterophylla*, during which the latter had a relatively small population size (*N*_e_ = 18 529). This phenomenon was largely due to the geographic isolation in glacial refugia. The divergence between *C. kweichowensis* and *C. heterophylla* was accompanied with constant and symmetric gene flow (m = 2.273). Hereafter, significant population expansions were observed in all three species, with the *N*_e_ values of *C. yunnanensis*, *C. kweichowensis*, and *C. heterophylla* estimated to be 32 439, 34 186, and 33 023, respectively (Fig. 3B). These population dynamics further led to the secondary contact of *C. kweichowensis* and *C. yunnanensis*, causing symmetric gene flow between the two adjacent species (m = 2.187). Based on the topological trios (((P1,P2)P3)O), the ABBA-BABA statistics revealed significant introgression signal between *C. kweichowensis* (P2) and *C. yunnanensis* (P3) (*D*-statistics = 0.059, *f*_4_-ratio = 0.17, *P* < 0.01) (Table S4, see online supplementary material), whereas no introgression was detected between *C. heterophylla* (P1) and *C. yunnanensis* (P3).

### Distributional shifts and habitat differentiation

Ecological niche modeling (ENM) was constructed to predict the current and past distributions for four evolutionary clades. All models displayed high predictive ability with area under the curve (AUC) values greater than 0.95. Jackknife tests of ENMs showed that precipitation of driest month (bio14), temperature seasonality (bio4), temperature seasonality (bio4), and annual precipitation (bio12) had the highest predictive contribution of 42.9%, 31.1%, 26.7%, and 36.9% for *C. avellana*, *C. heterophylla*, *C. kweichowensis*, and *C. yunnanensis*, respectively. The predicted ecological spaces of the four species were basically consistent with their actual distributions. During the glacial period from last interglacial (LIG) to last glacial maximum (LGM), *C. avellana* retreated to southern refuges due to extensive ice sheets in northern Europe, and then recolonized northern regions during the warm interglaciation from LGM to middle Holocene (MH). Within the Asian hazelnut complex, both *C. heterophylla* and *C. kweichowensis* showed clear southward range shifts during the transition from LIG to LGM, while *C. yunnanensis* exhibited significant northward expansion. This phenomenon is in line with the characteristics of global cold in northern China and favorable climate in southwest China during glacial periods. Since the MH period, all the four lineages have experienced population recovery or expansion, and basically formed their distribution patterns similar to the current predications (Figure S5, see online supplementary material).

**Figure 4:**
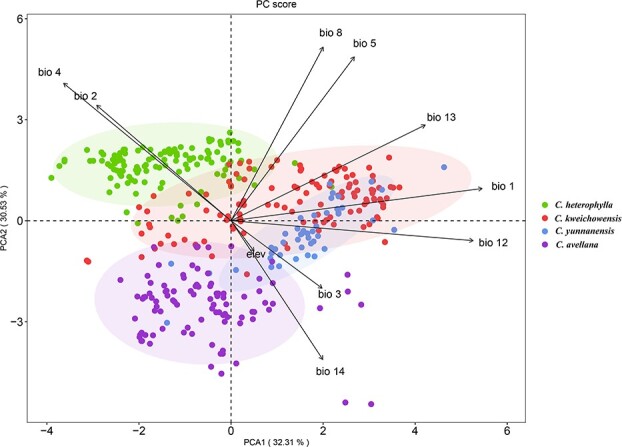
Habitat differentiation among four genetic lineages (*Corylus heterophylla*, *Corylus kweichowensis*, *Corylus yunnanensis*, and *Corylus avellana*) evaluated by environmental data. The biplot depicts the eigenvalues and the lengths of eigenvectors for PCA and each species is represented by a distinct color.

PCA analysis of environmental data indicated strong habitat differentiation among the four species (Fig. 4). The hierarchical clustering revealed four partially overlapped but largely distinct clusters of environmental variables, with significant ecological differences occurring at PC1 (32.31%) and PC2 (30.53%), respectively. The results of niche overlap and identity tests revealed obvious niche differentiation among pairwise species, with the observed values of *D* and *I* significantly lower than null distributions (Table S5, see online supplementary material). In partiuclar, there were remarkable ecological differences between *C. avellana* and the three geo-ecotypes of *C. heterophylla* complex, with *D* and *I* value ranging from 0.041 to 0.158, 0.133 to 0.388, respectively. Within the *C. heterophylla* complex, high niche differentiation was discovered between *C. heterophylla* and *C. yunnanensis* (*D* = 0.100, *I* = 0.286) (allopatric distribution), whereas slight overlap existed between parapatric species such as *C. kweichowensis* and *C. heterophylla* (*D* = 0.262, *I* = 0.544) or *C. yunnanensis* (*D* = 0.280, *I* = 0.568).

### Selection signals of geographic adaptation within *C. heterophylla* complex

**Figure 5:**
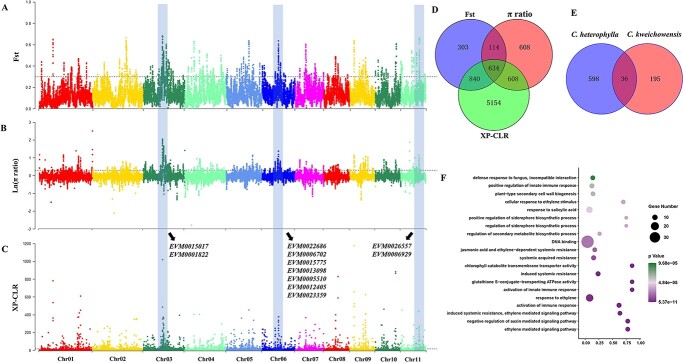
Selection sweep signals and candidate positively selected genes (PSGs) in *Corylus heterophylla*. **A** Manhattan plots for *F*_ST_ values between *C. heterophylla* and *Corylus kweichowensis*. **B** Manhattan plots for Ln (π ratio) values calculated by *C. kweichowensis / C. heterophylla*. **C** Manhattan plots for XP-CLR values between *C. heterophylla* and *C. kweichowensis*. The horizontal dashed lines indicate the significance threshold (5%) for selection signals. The grey columns represent the selection regions identified jointly by all three metrics (*F*_ST_, π ratio, and XP-CLR), with representative PSGs listed beside them. **D** Venn diagram of genes under selection detected using the *F*_ST_, π ratio, and XP-CLR methods. **E** Venn diagram of shared PSGs between *C. heterophylla* and *C. kweichowensis*. **F** GO enrichment scatter plot of PSGs in *C. heterophylla*.

**Figure 6:**
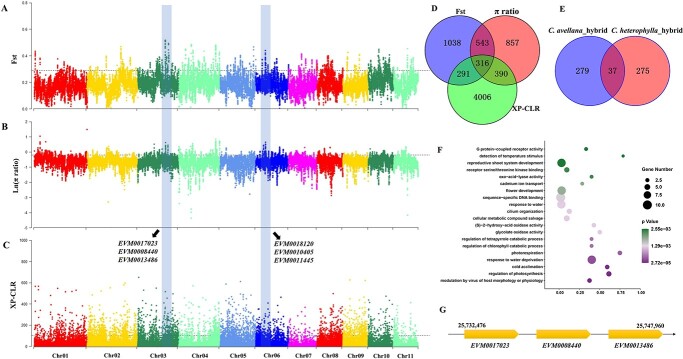
Selection sweep signals and positively selected genes (PSGs) in hybrid hazelnut. **A** Manhattan plots for *F*_ST_ values between hybrid hazelnut and *Corylus avellana*. **B** Manhattan plots for Ln (π ratio) values calculated by *C. avellana* / hybrid hazelnut. **C** Manhattan plots for XP-CLR values between hybrid hazelnut and *C. avellana*. The horizontal dashed lines indicate the significance threshold (5%) for selection signals. The grey columns represent the selection regions identified jointly by all three metrics (*F*_ST_, π ratio, and XP-CLR), with representative PSGs listed beside them. **D** Venn diagram of genes under selection detected using the *F*_ST_, π ratio, and XP-CLR methods. **E** Venn diagram of shared PSGs between *C. avellana*_hybrid and *Corylus heterophylla*_hybrid. **F** GO enrichment scatter plot of PSGs in hybrid hazelnut. **G** Three linked PSGs involved in cold acclimation and response to water deprivation.

The three geo-ecospecies of *C. heterophylla* complex have adapted locally through long-term selection under local environments. To identify genomic loci that favor local adaptation, we applied an integrative approach (*F*_ST_, *π* ratio, and XP-CLR; see ‘Materials and methods’) to detect signatures of selective sweeps for each species (Figs 5 and 6A–C; Figs S6–S9, see online supplementary material). The three metrics identified a total of 279 genomic regions (634 positively selected genes, PSGs) in *C. heterophylla* and 111 regions (231 PSGs) in *C. kweichowensis*, respectively (Fig. 5D and E; Table S6 and S7, see online supplementary material), when compared to each other. Meanwhile, 164 genomic regions harboring 446 PSGs were detected when *C. heterophylla* (low-altitude species) was compared to *C. yunnanensis* (high-altitude species) (Fig. S5; Table S6 and S7, see online supplementary material). Selection on these genes may underlie the genetic basis for different species to adapt to heterogeneous environments. We noted that few genes were shared between species pairs, suggesting the unique adaptive patterns for each species.

GO annotation was performed to explore the biological functions of these candidate genes. For *C. heterophylla*, the most significantly enriched terms included ethylene mediated signaling pathway, induced systemic resistance, and activation of innate immune response (*P* < 0.05) (Fig. 5F; Table S8, see online supplementary material). We found that a gene cluster of 7 PSGs on Chr06 (position: 10.73–10.86 Mb) functioned crucially in these biological processes, including EVM0006702, EVM0015775, EVM0013098, EVM0005510, EVM0012405, EVM0023359. Besides, several key PSGs – such as EVM0015017 and EVM0001822 on Chr03, EVM0026557 and EVM0006929 on Chr11 – were involved in regulation of secondary metabolite biosynthetic process. For *C. kweichowensis*, the highly enriched terms included regulation of Wnt and ethylene-activated signaling pathway, and regulation of flower/shoot system development (Table S9, see online supplementary material). Visibly, the response to ethylene stimulus has participated in important biological regulation in both *C. heterophylla* and *C. kweichowensis*. However, the PSGs involved varied greatly, with another gene cluster of 4 PSGs (EVM0022801, EVM0018078, EVM0009342, EVM0000178) on Chr11 (position: 1.02–1.06 Mb) for the latter ( Figure S6, see online supplementary material). For *C. yunnanensis*, 126 GO terms were significantly enriched with the keys involved in auxin transport, regulation of hormone levels, and histone H4 acetylation. Particularly, high-altitude selection signals like response to ionizing radiation, homologous recombination, and DNA repair were also detected (Table S10, see online supplementary material). Three key PSGs, EVM0015308, EVM0000300, and EVM0003598, were primarily responsible for these processes (Fig. S7, see online supplementary material).

### Selection and improvement signatures with hybrid combination

We performed a unidirectional comparison (*C. heterophylla*/*C. avellana*) to explore the adaptive signals of *C. avellana* under the European Mediterranean climate. The three selection tests (*F*_ST_, *π* ratio, XP-CLR) together identified 59 overlapped genomic regions and 109 PSGs (Tables S6 and S7, see online supplementary material), which should be credible candidate genes to contribute to the climate adaptation of *C. avellana*. Several significantly enriched terms were mainly involved in RNA processing, regulation of mitochondrial membrane potential, and divalent metal ion/inorganic cation transport (Table S11, see online supplementary material). We found that five PSGs (EVM0024746, EVM0003741, EVM0021897, EVM0019089, and EVM0018871) on Chr05, 07, and 10 were critically involved in RNA processing processes (Fig. S8, see online supplementary material), such as mRNA cleavage, mRNA metabolic process, and ncRNA catabolic process, etc.

Interspecific hybridization between *C. heterophylla* and *C. avellana* produced excellent hybrid cultivars, which not only exceed the commercial traits of *C. heterophylla*, but also expand more suitable regions than *C. avellana*. We conducted two unidirectional comparisons (*C. heterophylla*/hybrid and *C. avellana*/hybrid) to probe into the improvement signatures of hybrid hazelnut relative to its parents. The three methods combined allowed us to identify a total of 161 genomic regions (312 PSGs) in hybrid hazelnut when compared to its female parent *C. heterophylla*, and 124 genomic regions (316 PSGs) when compared to its male parent *C. avellana* (Fig. 6A–D; Table S5, see online supplementary material), of which 37 PSGs were overlapped (Fig. 6E; Table S6, see online supplementary material). For *C. heterophylla*/hybrid, 203 GO terms were significantly enriched and the key biological processes were mainly involved in regulation of pH, regulation of biological quality, and homeostatic process (Table S12, see online supplementary material). In particular, the regulation of biological quality should contribute to the quality attribute improvement of hybrid hazelnut, with the sub-process “regulation of protein polymerization and depolymerization” playing an essential role. Among the 22 PSGs involved in quality regulation, four (EVM0016084, EVM0017757, EVM0015097, and EVM0001784) predominantly participated in this process (Fig. S9, see online supplementary material). For *C. avellana*/hybrid, the PSGs were mainly involved in the biological processes of modulation by virus of host morphology or physiology, regulation of photosynthesis, cold acclimation, and response to water deprivation (Fig. 6F; Table S13, see online supplementary material), indicating significant improvement signals on resistance and adaptation. Three linked PSGs on Chr06 (EVM0011445, EVM0018120, EVM0010405) and three on Chr03 (EVM0017023, EVM0008440, EVM0013486) played major regulatory roles in the above processes (Fig. 6G).

## Discussion

The Section *Phyllochlamys* of genus *Corylus* represents the main body of hazelnut breeding in the world, with three closely related taxa (*C. avellana*, *C. americana*, and *C. heterophylla* species complex) and their hybrids (*C. avellana* × *C. heterophylla*, *C. avellana* × *C. americana*) widely distributed and cultivated in three continents. In this study, we focused on the eurytopic *C. heterophylla* complex in East Asia, the most commercially valuable *C. avellana* in Europe, and the improved hybrid hazelnut (*C. avellana* × *C. heterophylla*) in Mainland China, to investigate the genetic basis of evolution, adaptation, and improvement signatures in the intercontinental scale. The results could provide a valuable resource for research of hazelnut evolutionary biology and breeding, especially in terms of enhancing stress resistance.

### Evolutionary patterns of different genetic lineages

The taxonomic status of the three ecotypes (*C. heterophylla*, *C. kweichowensis*, and *C. yunnanensis*) within *C. heterophylla* complex has long been unsolved, with several controversial treatments proposed: (i) both *C. kweichowensis* and *C. yunnanensis* were the botanical varieties of *C. heterophylla* [10]; (ii) *C. yunnanensis* was treated as a distinct species, while *C. kweichowensis* was the variety of *C. heterophylla* [39]; and (iii) the three were all distinct species [21, 40]. However, these conclusions were reached based on limited diagnostic morphologies and traditional molecular markers that were insufficient to clarify the evolutionary relationships among sibling species. Based on multiple analyses (ML tree, PCA, and ADMIXTURE analysis), our study confirmed that these three geo-ecotypes were distinct species, which validated the third hypothesis at the population genomic level. In particular, the sister relationships between *C. heterophylla* and *C. kweichowensis* were supported by phylogenetic inference and low differentiation index. Besides, demographic simulation revealed that *C. yunnanensis* was the first to split from the species complex, while *C. kweichowensis* and *C. heterophylla* diverged from each other at a more recent scale (Fig. 3B). From the perspective of evolution, these three lineages may represent the early stage of parapatric speciation, during which gene flow will occur frequently between diverged species. This point is evidenced by the significant migration rates between pairwise adjacent species: *C. kweichowensis* and *C. yunnanensis*, *C. kweichowensis* and *C. heterophylla*. Under this circumstance, divergent ecological selection is supposed to maintain genetic distinction in the face of ongoing gene flow. Such ecological speciation has also been detected in population genomic studies of other plants, such as *Buddleja alternifolia* [41], *Populus rotundifolia* [42], and *Salix brachista* [43]. Still, a transient interruption of gene flow between *C. yunnanensis* and ancestors of *C. kweichowensis* and *C. heterophylla* was detected. This was largely caused by geographic isolation in glacial refugia because the latter had a relatively small population size (via glacial contraction) at that time. However, the combination of *N*_e_ dynamics (Fig. 3), distribution shifts (Fig. 4), and ABBA-BABA statistics (Table S4, see online supplementary material) demonstrated that population expansions have led to secondary gene flow and introgression between *C. kweichowensis* and *C. yunnanensis*.

Evaluation of genetic relationships among germplasms can provide theoretical basis for hybrid breeding. Due to excellent commercial properties, *C. avellana* has always been used as a candidate parent for hazelnut improvement in other regions. Accordingly, we investigated the genome-level differentiation between *C. avellana* and *C. heterophylla* complex. Our results revealed evident interspecific differentiation between *C. avellana* and any of these three species (average *F*_ST_ = 0.323), with the *F*_ST_ values ordered as *C. yunnanensis* > *C. heterophylla* > *C. kweichowensis* (Fig. 2B). Despite all this, it has been proved that *C. avellana* can easily hybridize with the three species without genetic barriers [16], which implies that reproductive isolation has not yet developed among these related species. Typically, the emergence and promotion of hybrid hazelnut in northern China represent a successful practice of interspecific hybridization. Moreover, the similar historical trajectory for *C. avellana*, *C. heterophylla*, and *C. kweichowensis* (Fig. 3A) indicates that the three have a shallow divergence time. Taken together, the high-level genetic differentiation between *C. avellana* and Asian sibling species should be shaped by recent demographic and selective processes, such as Quaternary glaciation and domestication, which can affect diversity and differentiation level in a shorter time. Homoploid hybridization among closely related species has been recognized as an important mechanism for speciation [26, 44, 45]. Although hybrid hazelnut inherited mixed genetic components from *C. avellana* and *C. heterophylla*, it was determined as a distinct lineage by admixture analysis (Fig. 1C). Particularly, hybrid hazelnut showed the highest genetic diversity and heterozygosity among the five genetic lineages, indicating that hybridization can promote the accumulation of nucleotide polymorphisms.

### Local adaptation of *C. heterophylla* complex

For long-term sedentary plants, adaptive evolution is the fundamental process for their populations to respond to diverse environments [46, 47]. Exploring genetic variations associated with adaptation can provide valuable resources for germplasm improvement. The three geo-ecospecies of *C. heterophylla* complex have adapted locally through long-term selection under local environments. *C. heterophylla* occurs extensively in dry and cold temperate of northern China at an altitude of 200 ~ 1000 m, and *C. kweichowensis* grows vigorously in sticky and high humidity subtropics of central and eastern China at an altitude of 700 ~ 2500 m, whereas *C. yunnanensis* is narrowly distributed in eastern QTP of southwest China with a high altitude of 2000 ~ 3700 m. Consistent with this, habitat differentiation analysis demonstrated that ecological selection may have facilitated the adaptive divergence of the three species (Fig. 4; Table S5, see online supplementary material). Accordingly, we applied three genome-wide metrics to detect signatures of selective sweeps for each species. We found PSGs involved in specific functions such as regulation of signaling pathway, development, and resistance have contributed to the divergence between *C. heterophylla* and *C. kweichowensis* (Tables S7 and S8, see online supplementary material). In particular, the response to ethylene stimulus participated in some key biological processes in both species. It is reasonable because ethylene regulates multiple development and physiological processes, such as seed germination, cell development, flowering, fruit ripening, response to biological and abiotic stress, etc [48]. Even so, we found that few PSGs were shared among the two species (Fig. 5E; Table S7, see online supplementary material), indicating the unique adaptive patterns for each species and that different climates may shape distinct genomic regions. *C. yunnanensis* is an alpine tree in eastern QTP and occupies almost the highest habitats in the genus *Corylus*. Alpine plants are exposed to multiple abiotic stimuli, including strong UV radiation, low temperature, and long sunshine [49–51], which could easily lead to DNA and cell damage [52]. Correspondingly, DNA and cell repair after damage are vital for the high-altitude adaptation of alpine plants. As expected, we identified several significantly enriched terms involving in response to ionizing radiation, homologous recombination, and DNA repair (Fig. S7; Table S10, see online supplementary material), suggesting the unique adaptation of *C. yunnanensis* to high-altitude environment. Overall, although harsh environments have posed strong challenges to the survival of hazelnuts, the adaptive variations generated under stress pressure can offer excellent resources for breeding.

### Selection and improvement signatures within hybrid combination

After long generations of selection and domestication, *C. avellana* has obtained excellent agronomic traits that other species cannot match. However, its promotion in Mainland China is restricted due to climate inadaptation. Fortunately, interspecific hybridization between *C. avellana* and *C. heterophylla* produced excellent hybrid cultivars that are widely cultivated in northern China. This hybrid combination not only provides an ideal model to test how sibling species adapt to contrasting environments, but also can detect the improvement signatures of hybrids relative to the parent species. We emphatically explored the adaptive divergence between *C. avellana* and *C. heterophylla* and found that the PSGs were mainly involved in RNA 3′-end processing, mRNA cleavage, and ncRNA catabolic process (Table S11, see online supplementary material). These RNA processing processes can affect the expression level of specific genes, thus ensuring the normal development of plants and improving the adaptation to environmental changes [53, 54]. Therefore, these PSGs should have important contributions to the adaptation of *C. avellana* to the Mediterranean climate. In addition, we also found that PSGs regulating mitochondrial membrane potential and metal ion transport were also significantly enriched, which is consistent with the local soil characteristics. The Mediterranean region is covered with red-brown soils rich in free iron oxide and other metallic elements, which may impose specific selection on *C. avellana* compared to other regions. Interspecific hybridization can generate novel allelic combinations, thus leading to new phenotypes and genotypes [55, 56]. Hybrids are usually superior to both parents in one or multiple traits (e.g. growth vigor, fruit size, and quality), which can be viewed as an improvement stage of selection breeding. In practice, hybrid hazelnut inherited not only the commercial traits (large nut with thin shells and uniform shape) of *C. avellana*, but also the stress resistance (cold and drought) of *C. heterophylla*. In line with this, we detected PSGs associated with improvement in hybrid hazelnut, including those that regulate protein polymerization (e.g. *EVM0015097* and *EVM0001784*) and photosynthesis (e.g. *EVM0011445*; *EVM0018120*; *EVM0010405*) (Tables S12 and S13, see online supplementary material), which significantly influence growth capacity and reproduction. Besides, we also identified PSGs that are related to stress resistance, such as those involved in cold acclimation (e.g. *EVM0017023*) and response to water deprivation (e.g. *EVM0008440* and *EVM0013486*). These findings are compatible with the improved traits expected to be influenced by selection breeding in hybrid hazelnut.

## Materials and methods

### Population sampling and genome resequencing

A total of 125 diverse accessions within Section *Phyllochlamys* were collected from the hazelnut germplasm repository of Research Institute of Forestry, Chinese Academy of Forestry (Beijing, China), including 32 *C. heterophylla*, 25 *C. kweichowensis*, 19 *C. yunnanensis*, 12 *C. avellana*, and 37 hybrid cultivars. The 76 accessions of the three geo-ecotypes within Asian hazelnut complex basically cover the natural distribution regions in northern China, central and eastern China, and southwest China. The 12 *C. avellana* accessions represent six domesticated breeds widely cultivated in most European countries, and the 37 hybrid cultivars have been identified to have wider suitable regions than their female parent (*C. heterophylla*) in northern China (Figure S1; Table S1, see online supplementary material). The genomic DNA of the 125 hazelnut accessions were extracted using the DNeasy Plant Mini Kit from Qiagen, Inc. (Berlin, Germany) and then used to construct 400-bp insert pair-end (PE) libraries at Berry Genomics Co. Ltd. (Beijing, China). Subsequently, the libraries were sequenced on an Illumina NovaSeq6000 platform with 150 bp paired-end reads. All individuals were sequenced to a target depth (20×) and a total of 1419 Gb raw data were obtained.

### Read mapping, variant calling, and annotation

The raw reads were filtered using Trimmomatic v0.36 [57] to remove low-quality reads and adapter sequences with default parameters. All clean reads were mapped to the *C. heterophylla* reference genome [30] using BWA-MEM v0.7.16 [58] with default options. Then, SAMtools v0.1.19 [59] was used to convert the mapping results to BAM format, and Picard v1.117 (https://broadinstitute.github.io/picard/) was employed to remove PCR duplicates. The mapping rate, sequencing depth, and coverage rate were calculated with the DepthOfCoverage program in GATK v4.0.5.1 [60]. Single nucleotide polymorphism (SNP) calling was performed in GATK v4.0.5.1 using HaplotypeCaller and GenotypeGVCFs tools. In order to reduce the false positives of variant detection, a strict filtration was applied with the following criteria: (i) both mapping quality and base quality <20; (ii) extremely low (< one-third average depth), or extremely high (> twofold average depth) coverage; (iii) minor allele frequency < 0.05 and missing rates >20%. Captured variants were merged with GenotypeGVCFs. To avoid the effect of excessive linkage on population analysis, SNP sites were LD-pruned in PLINK v 1.90 [61] with the parameter “-indep-pairwise 50 10 0.2”. Finally, a total of 7 177 893 SNPs were annotated with Snpeff v4.3 [62] based on the annotation of the *C. heterophylla* reference genome [35]. The final SNPs were categorized in coding regions, intergenic regions, exonic regions, intronic regions, and splicing sites. SNPs in coding regions were further divided into synonymous and non-synonymous types. An SNP density map was drawn using the CMplot package in R.

### Phylogenetic inference and population structure

We produced a maximum likelihood (ML) tree based on the LD-pruned SNP dataset (7177893), as implemented by RaxML v8.2.12 [63]. The ML tree was constructed using the gamma model of rate heterogeneity with the Lewis ascertainment bias (ASC_ GTRGAMMA). One hundred rapid bootstraps and ML search for the optimal scoring tree were conducted in a single run. The phylogenetic tree was visualized using the online program iTOL (https://itol.embl.de/). Principal component analysis (PCA) was performed using GCTA v1.26 [64] and the first three eigenvectors were plotted in two dimensions. We also investigated the population structure using ADMIXTURE v1.3.0 [65] based on the LD-pruned SNP dataset, setting the postulated group numbers (*K*) from 2 to 8 and each running 10 000 iterations. The optimum number of clusters (*K*) was determined based on the minimum cross-validation (CV) error value.

### Genetic diversity and differentiation analyses

To compare the genetic diversity and genetic differentiation among different species, nucleotide diversity (*π*) values and pairwise fixation index (*F*_ST_) were calculated for each locus using VCFtools v0.1.13 [66]. A sliding window of 100 kb with a 10 kb step size across the *C. heterophylla* genome was assigned for window calculation of *π* and *F*_ST_. The interaction figure of *π* and *F*_ST_ values of different species was drawn by Cytoscape v3.5.1 [67]. Besides, heterozygosity rate of each accession was evaluated using the program PLINK v1.90 [61]. Heterozygosity was the quotient of the number of heterozygous SNPs divided by total genome length.

### Linkage disequilibrium analysis

To measure the LD level, the squared correlation coefficient (*r^2^*) was calculated for pairwise SNPs along 500 kb windows using the PopLDdecay v3.31 [68] in each species with the following parameters: -MaxDist 500 -MAF 0.05 -Miss 0.2 -OutFilterSNP -OutPairLD 1. The decayed physical distance between SNPs was identified as the distance at which the maximum *r^2^* dropped by half.

### Demographic history and gene flow

The trajectory of effective population size (*N*_e_) for four evolutionary clades (*C. heterophylla*, *C. kweichowensis*, *C. yunnanensis*, and *C. avellana*) was inferred by the pairwise sequentially Markovian coalescent (PSMC) model [69]. Because PSMC has high false-negative rates at low sequencing depth, we limited this analysis to four samples with the highest coverage (≥20×) in each taxa to ensure the quality of consensus sequences. PSMC analysis was set with the following parameters: -N25 -t15 -r5 -p “4 + 25 × 2 + 4 + 6”. A neutral mutation rate (*μ* = 3.75 × 10^−8^ per base per generation) and average generation time (*g =* 15 years) were applied to convert the population sizes and scaled times into real sizes and times [70].

In order to explore the intracontinental divergence pattern, we further used ∂a∂i to estimate the demographic histories of *C. heterophylla* complex under different divergence scenarios. Based on a coalescent framework, ∂a∂i predicts the site frequency spectrum (SFS) of genetic variation among populations enabling statistically rigorous assessments on population size, migration rates, and divergence times [71]. We tested five three-population divergence scenarios and 17 demographic models (Figure S2, see online supplementary material): (i) divergence with gene flow (models 1–4); (ii) Ancient Migration (models 5–7); (iii) secondary contact (models 8–10); (iv) simultaneous splitting (models 11–14); and (v) admixed (“Hybrid”) origin models (models 15–17). Because ∂a∂i is quite sensitive to missing data and distribution of allele frequencies, we thus generated a new subset of 3 804 556 SNPs by filtering all missing sites and retaining low-frequency sites across all samples. Two-dimensional folded SFS based on these sites was estimated by easySFS (https://github.com/isaacovercast/easySFS). Based on threefold randomly perturbed starting parameters, we conducted 50 optimized replicates using the Nelder–Mead method with a maximum of 20 iterations for each model included in the 3D model sets. The global maximum log-likelihood model was selected after correcting for number of estimated parameters using Akaike information criterion. To obtain 95% confidence intervals based on the best fitting parameters, simulation was carried out 100 times. The parameters estimated by ∂a∂i were scaled by *N*_e_ which was calculated through the formula *N*_e_ = θ/(4 *μL*), where *θ* represents the effective mutation rate of the ancestral population, *μ* is the mutation rate (3.75 × 10^−8^ per base per generation), and *L* is the genome size of *C. heterophylla* (370.75 M). The above parameters were then used to estimate the divergence time and population size.

To further explore introgression patterns between taxa of *C. heterophylla* complex, we performed Patterson’s *D*-statistic (ABBA-BABA statistic) and the related admixture fraction estimates (*f*_4_-ratio statistics) using Dsuite software [72]. *D*-statistic test was used to assess the imbalance between ABBA and BABA allele patterns. Tests were conducted with a four-taxon fixed phylogeny: given an outgroup *C. avellana* (O), we set three ingroup taxa of *C. heterophylla* (P1), *C. kweichowensis* (P2), *C. yunnanensis* (P3) with the relationship (((P1,P2)P3)O). Without introgression, conflicting ABBA and BABA patterns should arise with equal frequencies via incomplete lineage sorting, producing a *D*-statistic equal to 0. Whereas, if introgression between P1 or P2 and P3 has occurred, the ABBA pattern should be excessive relative to BABA, and the *D*-statistic deviates significantly from zero. First, we used the Dtrios program to calculate *f*_4_-ratio for all species based on the phylogeny; then, *f*-branch statistics were estimated for each phylogenetic branch by the Fbranch program; finally, we adopted the *f*-branch metric to tease apart potentially correlated *f*_4_-ratio statistics and identify introgression events between internal branches.

### Distributional shifts and habitat differentiation

To explore the patterns of distribution shifts within Section *Phyllochlamys*, we further conducted ENM to predict potential distribution for four evolutionary taxa at the current time, during the last glacial maximum (LGM, ~0.022 Mya), the last interglacial (LIG; ~0.14–0.12 Mya), and the middle Holocene (MH, ~0.006 Mya). Nineteen bioclimatic variables and one elevation layer used for ENM analysis were retrieved from the WORLDCLIM database (www.worldclim.org). To avoid multicollinearity, environmental variables were subjected to redundancy removal using a threshold for Pearson’s correlation coefficients (*r*) >0.75 [73]. Finally, 10 environmental variables were retained for subsequent analysis (Table S14, see online supplementary material). A total of 398 occurrence records were collected from herbarium records (http://www.gbif.org) and field investigations, including 100 *C. avellana*, 131 *C. heterophylla*, 121 *C. kweichowensis*, and 46 *C. yunnanensis* (Table S15, see online supplementary material), which covered nearly the whole natural ranges of these species. ENM analyses were performed based on the current variables and projected against the other two historical variables using the maximum entropy model in MAXENT v3.3.4 [74] with the parameters set as: type = subsample; replicates = 20; maximum iterations = 5000; random test points = 25. Changes in distribution shifts of these geo-ecospecies as revealed by ENM analysis would provide an environmental context for the effects of Quaternary climate change on their demographic history. Model accuracy was assessed using the AUC of the receiver operating characteristics (ROC).

To better elucidate the habitat differentiation within Section *Phyllochlamys*, we performed PCA based on 10 environmental variables and 400 occurrence records. The climate data were retrieved using ArcGIS 10.3 [75] for all these 398 geographical coordinates to form environmental matrix. Then, a two-dimensional scatter plot was constructed with Origin 9.1. In addition, we conducted niche overlap and identity tests to evaluate niche differentiation between species by computing the Schoener’s *D* and the standardized Hellinger distance (*I*) in ENMtools v1.4.3 [76]. The values of *D* and *I* (0–1) indicate the similarity or difference of the niches, which represent no overlap and completely identical, respectively.

### Genomic signals of during adaptation and improvement

To detect selection signals during geographic adaptation and improvement for the five species, selective sweep analyses were performed based on three genome-wide metrics, including genetic differentiation index (*F*_ST_), nucleotide diversity ratio (*π*_m_/*π*_n_, where *π*_m_ and *π*_n_ were the *π* values of pairwise species, respectively), and the cross-population composite likelihood ratio test (XP-CLR). On the basis of potential evolutionary scenario and breeding background, two types of selection signals were investigated. First, we explored the genetic footprints of local adaptation within the Asian *C. heterophylla* complex. For *C. heterophylla* and *C. kweichowensis*, reciprocal comparisons were performed to investigate their adaptive divergence caused by north–south climate differences. To identify positive selection signals involved in high-altitude adaptation of *C. yunnanensis*, we conducted a one-way comparison between lowland species (*C. heterophylla*) and highland species (*C. yunnanensis*) for identification. Second, we scanned the genomic signatures associated with adaptation and improvement within the hybrid combination (*C. avellana*, *C. heterophylla*, and hybrid cultivars). Taking Asia *C. heterophylla* as the control, we conducted a unidirectional comparison with *C. avellana* so as to explore the adaptive signals of the latter under the European Mediterranean climate. Moreover, two unidirectional comparisons (i.e. *C. heterophylla*/hybrid and *C. avellana*/hybrid) were implemented to identify the improvement signals of hybrid hazelnut relative to its parents. The statistics of *F*_ST_ and *π* ratio were calculated with 100-kb sliding windows and 10-kb steps across the genome using vcftools [66]. For XP-CLR analysis, we scanned the genome with 0.05-cM sliding windows and 100-bp grid steps, fixing the maximum number of SNPs assayed in each window to 200. Then, the mean likelihood scores were calculated with the same sliding windows and step sizes as *F*_ST_ and *π* ratio. The top 5% genomic regions identified by all the three metrics were designated as putative selective sweeps.

Functional analysis for candidate genes was performed against the Gene Ontology (GO) database [77]. Functional classification of GO categories was carried out utilizing the Blast2GO program [78]. Enrichment analysis was implemented with the TopGO package (Alexa and Rahnenfuhrer, 2010) and the chi-squared test was used to evaluate the statistical significance. We centered on those genes with significant GO terms (*P* < 0.05) in corresponding lineages.

## Supplementary Material

Web_Material_uhad065

## Data Availability

The raw sequencing data of 125 accessions have been submitted to the NCBI BioProject database under accession number PRJNA899594. The unfiltered variant call format (VCF) file relative to the resequencing experiment is publicly available at the Figshare repository (10.6084/m9.figshare.22306681).
