# Supplementary material for: Population genomics reveals demographic history and selection signatures of hazelnut (Corylus)
Source: Hortic Res. 2023 Apr 10;10(5):uhad065. doi: 10.1093/hr/uhad065 (PMC10208898; doi:10.1093/hr/uhad065)
Supplement: Web_Material_uhad065 [file web_material_uhad065.zip › Supplementary Figures.docx]

**Population genomics reveals demographic history and selection signatures in hazelnut species (Section *Phyllochlamys, Corylus*)**

**Supplementary Figures**


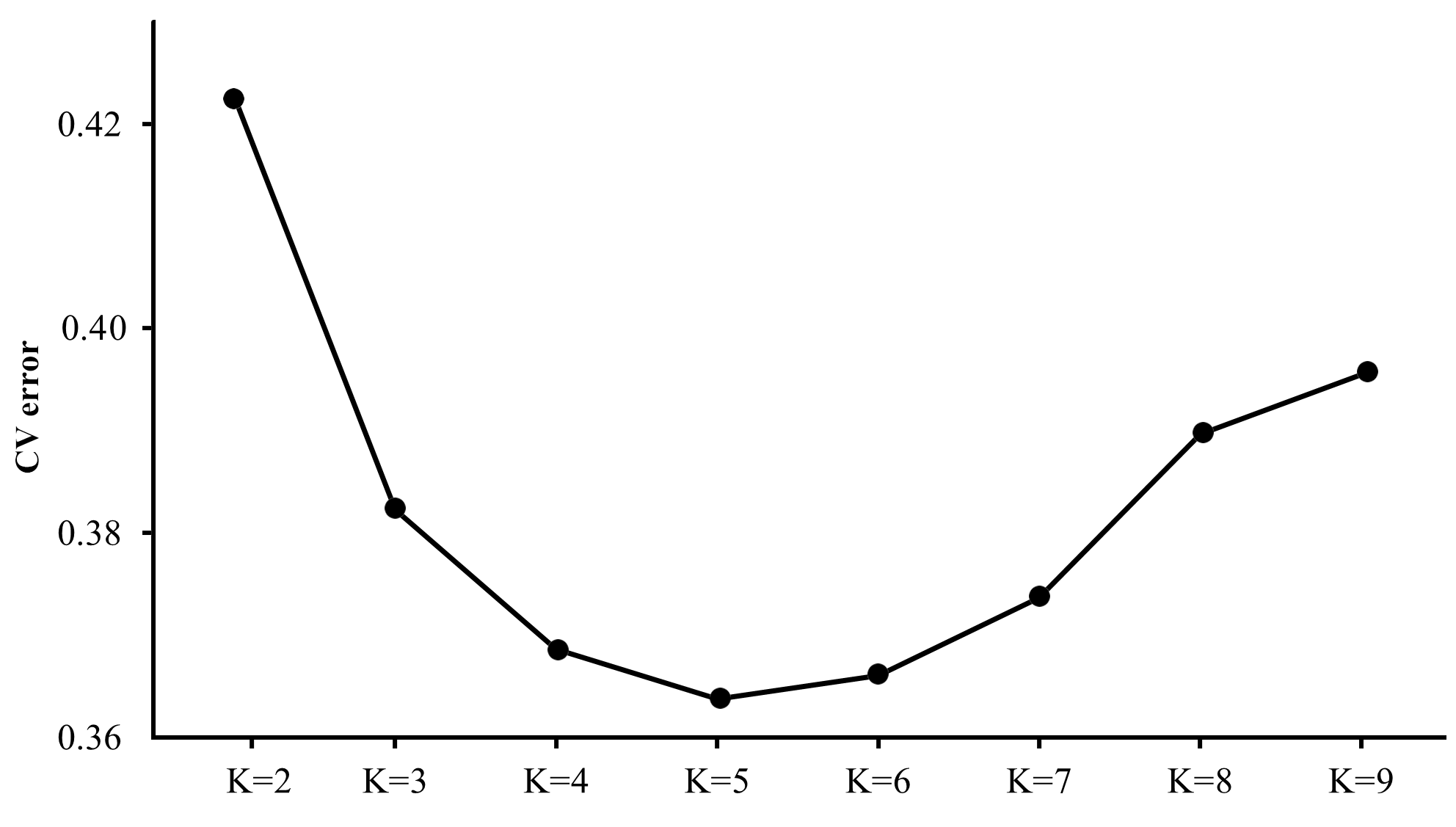


**Fig. S1.** Cross-validation plot (*K*=2-9) for hazelnut accessions in AMIXTURE analysis. The cross-validation error showed a nadir at *K*=5, suggesting five clusters as the optimal option.


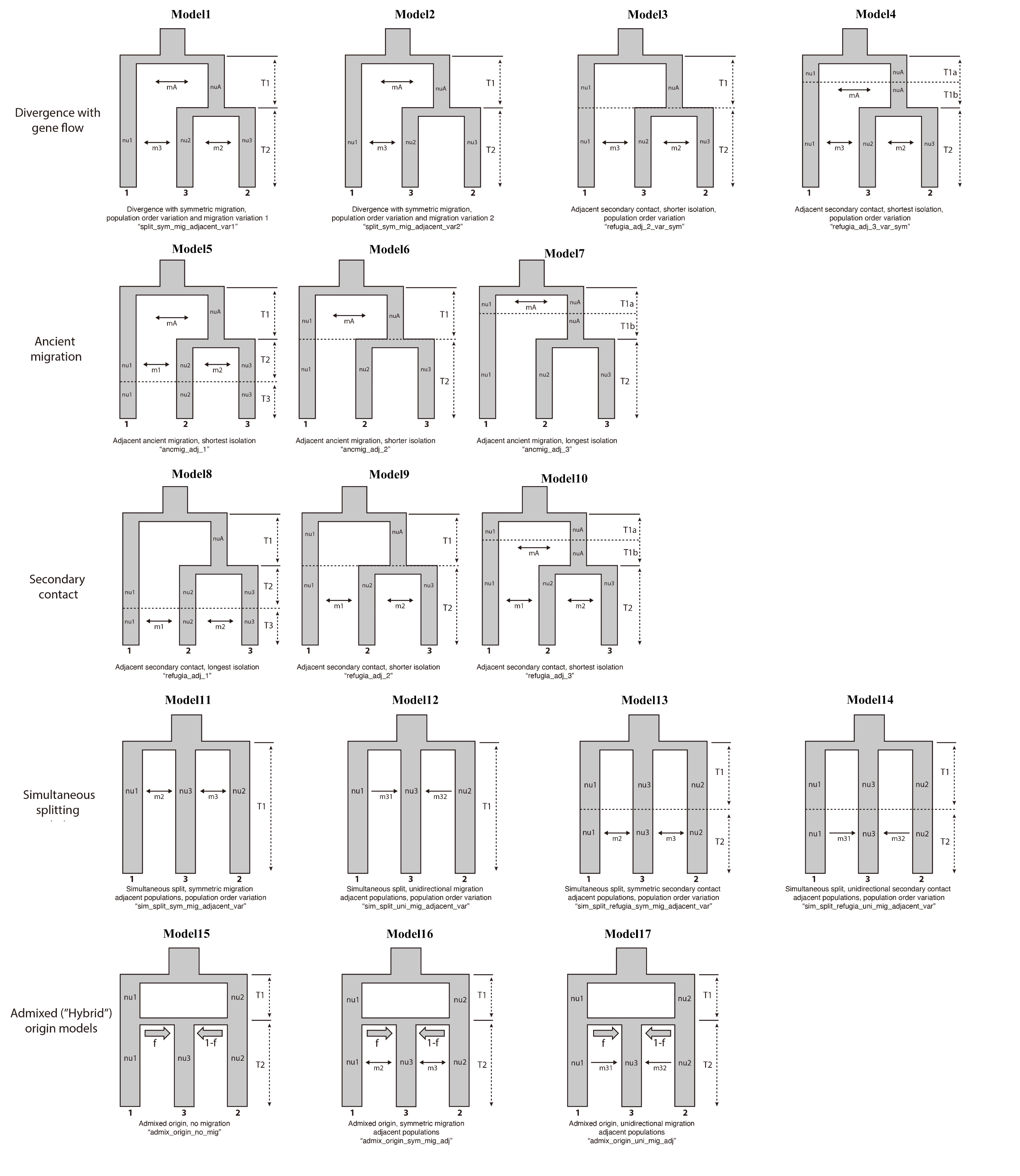


**Fig. S2.** Schematic representation of 17 three-population models in the analysis of demographic history. 1, 2 and 3 refer to the three subgroups (*C. yunnanensis*, *C. kweichowensis*, and *C. heterophylla*). T1a: The scaled time between the split of subgroups 1 vs 2 and 3 (in units of 2**N*_e_ generations). T1b: The scaled time for no gene flow between subgroups 1 and (2, 3) (in units of 2* *N*_e_ generations). nu1a: Size of subgroups 1 after split. nu2a: Size of subgroups 2 after split. nu3a: Size of subgroups 3 after split. nu1b: Size of subgroups 1 after size change. nu2b: Size of subgroups 2 after size change. nu3b: Size of subgroups 3 after size change. nuA: Size of subgroups (2, 3) after split from 1. mA: Migration rate between subgroups 1 and subgroups (2, 3). m1: Migration rate between subgroups 1 and 2 (2* *N*_e_ *m). m2: Migration rate between subgroups 2 and 3. m3: Migration rate between subgroups 1 and 3. T1: The scaled time between the split of subgroups 1 vs 2 and 3 (in units of 2*Ne generations). T2: The scaled time between the split of subgroups 2 and 3 (in units of 2**N*_e_ generations). T3: The scaled time between the size change and the present (in units of 2**N*_e_ generations).


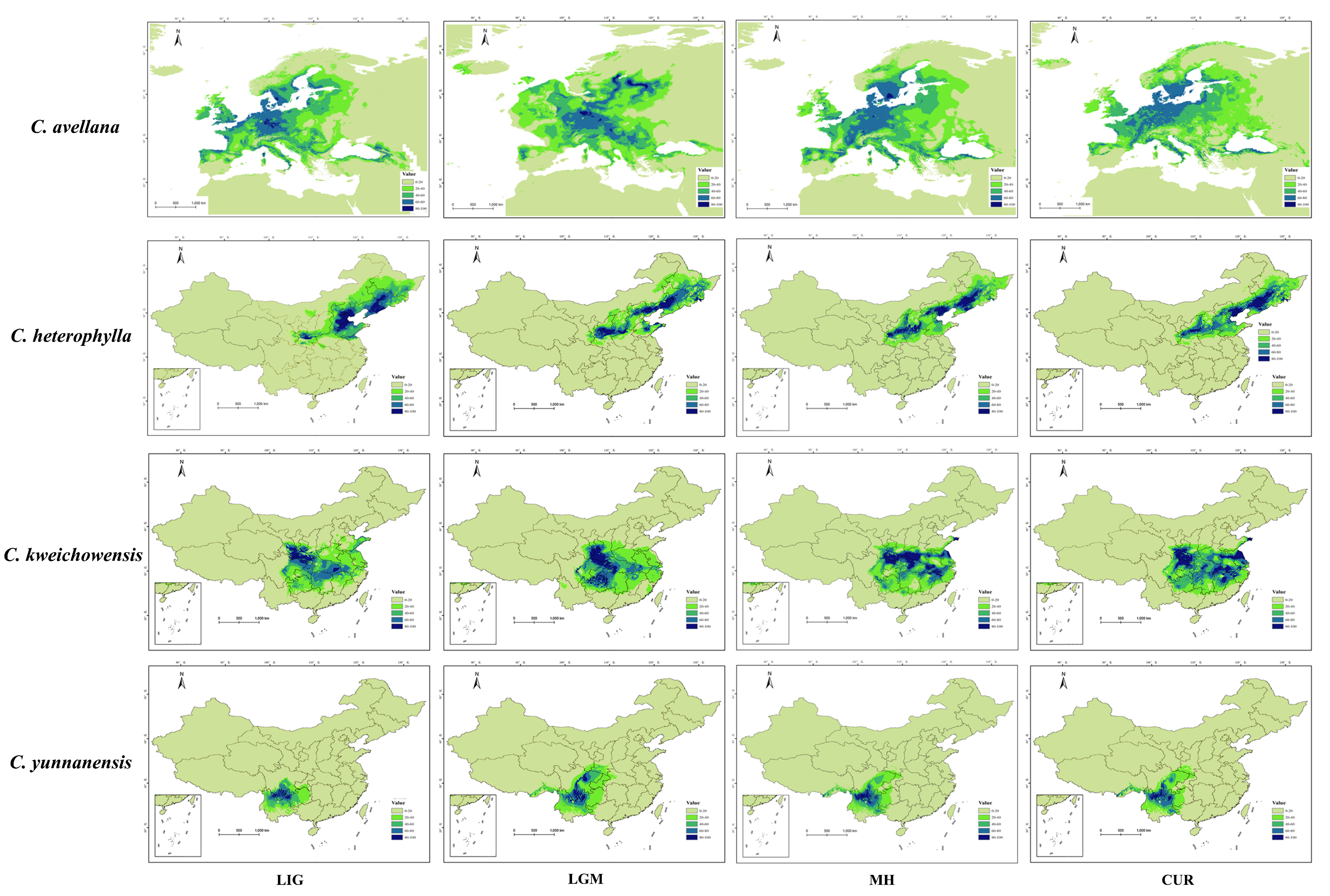


**Fig. S3.** Predicated distributions of four hazelnut clades in different periods based on ecological niche modeling. LIG: last interglacial; LGM: last glacial maximum; MH: middle Holocene; CUR: current.


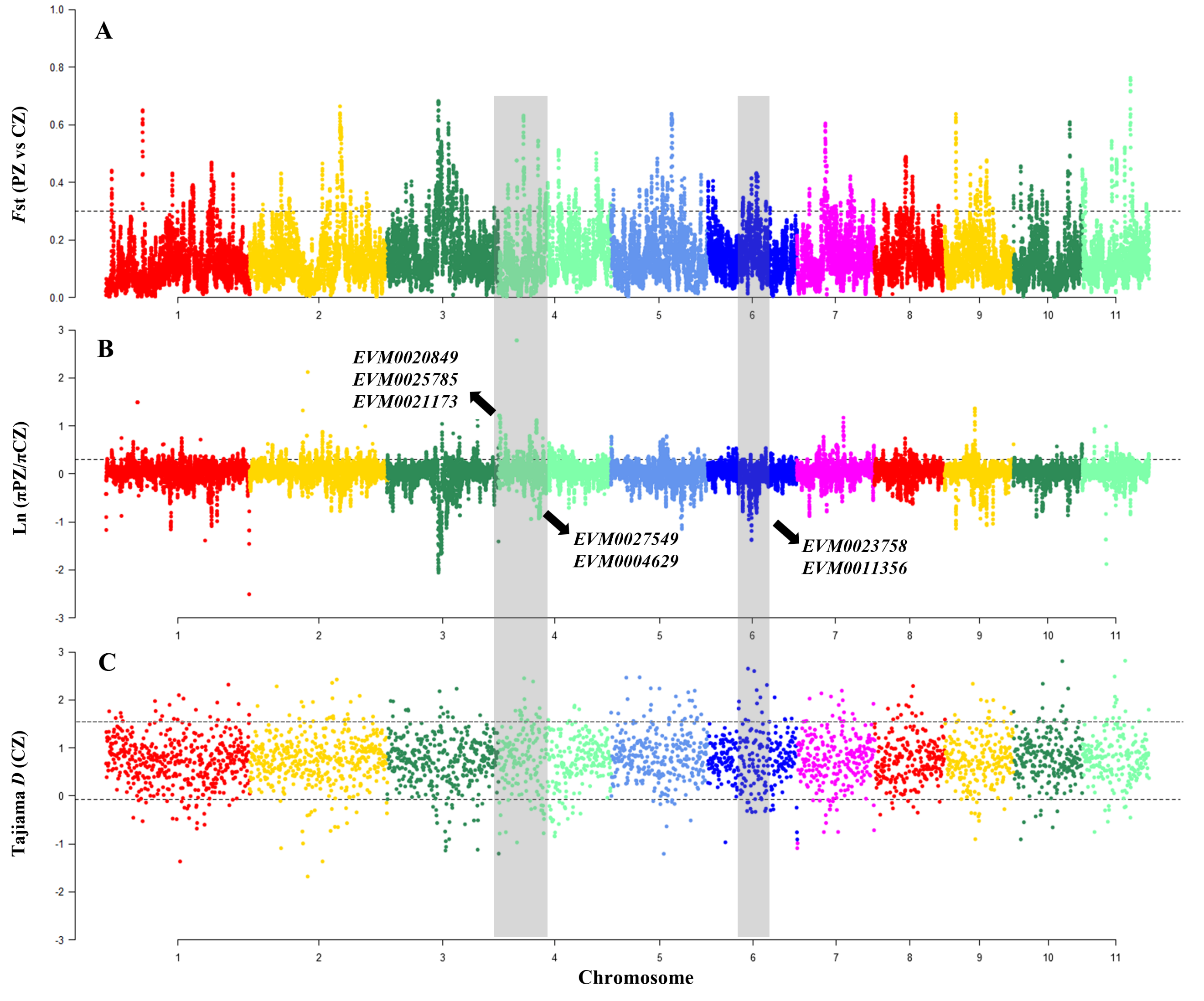


**Fig. S4**. Selection sweep signals and examples of positively selected genes (PSGs) in *C. kweichowensis*. (A) Highly divergent regions between *C. heterophylla* and *C. kweichowensis* measured by the top 5% of *F*_ST_. (B) Nucleotide diversity differences between *C. heterophylla* and *C. kweichowensis* measured by the top 5% of π ratio. (C) Selection signals identified by the top and tail 5% of Tajima’s *D* values in *C. kweichowensis*. The grey columns represent the selection regions identified jointly by all three metrics (*F*_ST_, π ratio, and Tajima’s *D*), with seven representative PSGs listed beside them. The dashed lines indicate the significance threshold (5%) of corresponding metrics.
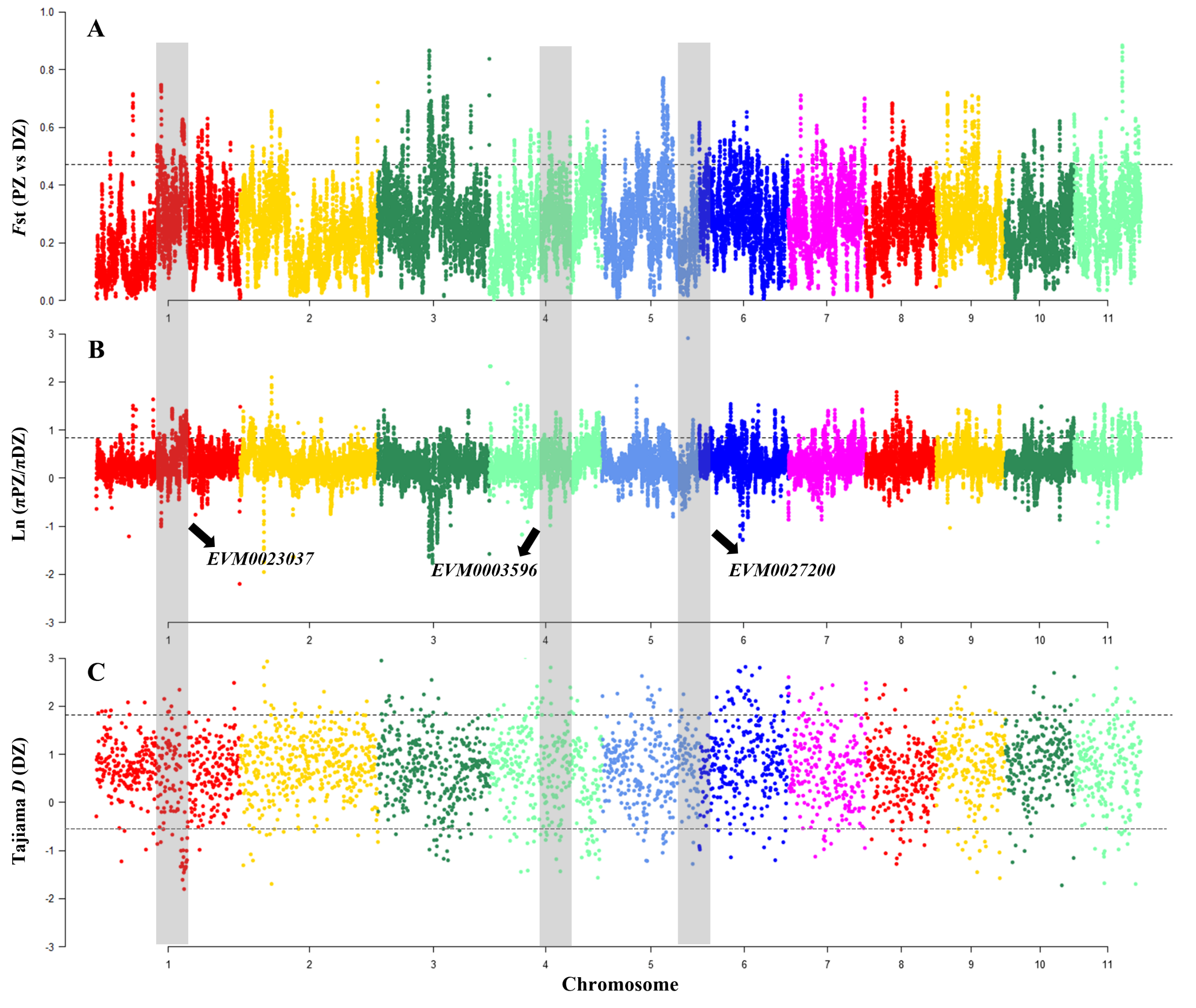


**Fig. S5**. Selection sweep signals and examples of positively selected genes (PSGs) in *C. yunnanensis*. (A) Highly divergent regions between *C. heterophylla* and *C. yunnanensis* measured by the top 5% of *F*_ST_. (B) Nucleotide diversity differences between *C. heterophylla* and *C. yunnanensis* measured by the top 5% of π ratio. (C) Selection signals identified by the top and tail 5% of Tajima’s *D* values in *C. yunnanensis*. The grey columns represent the selection regions identified jointly by all three metrics (*F*_ST_, π ratio, and Tajima’s *D*), with three representative PSGs listed beside them. The dashed lines indicate the significance threshold (5%) of corresponding metrics**.**
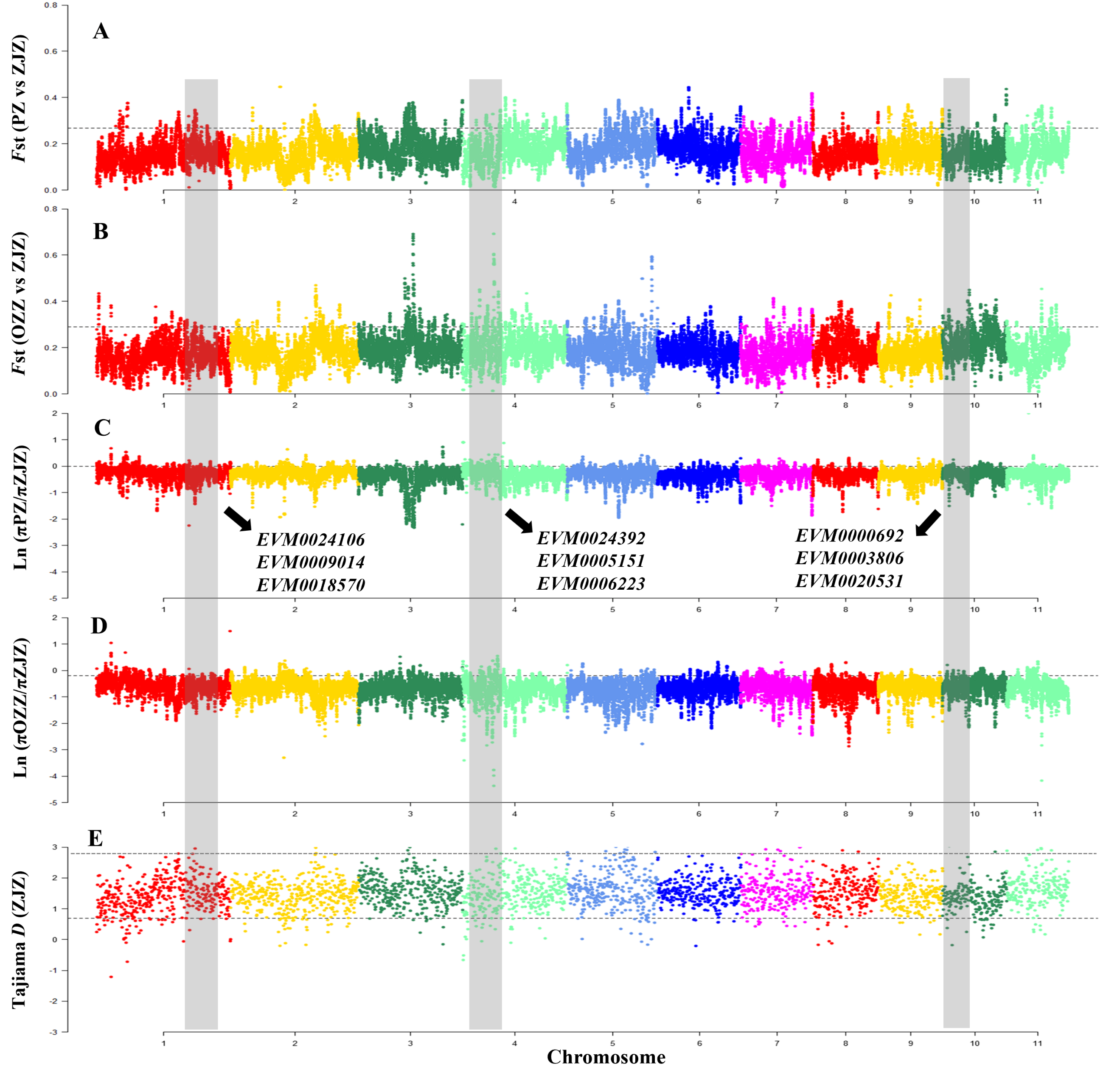


**Fig. S6**. Selection sweep signals and examples of positively selected genes (PSGs) in hybrid hazelnut. (A) Highly divergent regions between *C. heterophylla* and hybrid hazelnut measured by the top 5% of *F*_ST_. (B) Nucleotide diversity differences between *C. avellana* and hybrid hazelnut measured by the top 5% of π ratio. (C) Selection signals identified by the top and tail 5% of Tajima’s *D* values in hybrid hazelnut. The grey columns represent the selection regions identified jointly by all three metrics (*F*_ST_, π ratio, and Tajima’s *D*), with three representative PSGs listed beside them. The dashed lines indicate the significance threshold (5%) of corresponding metrics**.**
